# gofasta: command-line utilities for genomic epidemiology research

**DOI:** 10.1093/bioinformatics/btac424

**Published:** 2022-07-05

**Authors:** Ben Jackson

**Affiliations:** Institute of Ecology and Evolution, University of Edinburgh, Edinburgh EH9 3FL, UK

## Abstract

**Summary:**

gofasta comprises a set of command-line utilities for handling alignments of short assembled genomes in a genomic epidemiology context. It was developed for processing large numbers of closely related SARS-CoV-2 viral genomes and should be useful with other densely sampled pathogen genomic datasets. It provides functions to convert sam-format pairwise alignments between assembled genomes to fasta format; to annotate mutations in multiple sequence alignments, and to extract sets of sequences by genetic distance measures for use in outbreak investigations.

**Availability and implementation:**

gofasta is an open-source project distributed under the MIT license. Binaries are available at https://github.com/virus-evolution/gofasta, from Bioconda, and through the Go programming language’s package management system. Source code and further documentation, including walkthroughs for common use cases, are available on the GitHub repository.

## 1. Introduction

gofasta provides utilities that handle fasta-format alignments of viral consensus genomes in a genomic epidemiology context. Its development was motivated by the scale and nature of SARS-CoV-2 genome datasets, which are orders of magnitudes larger than any previous pathogen sequence dataset, which necessitates efficient and scalable analysis methods (Hodcroft *et al.*, 2021). gofasta is a command-line tool written in Go (https://go.dev) and is freely available under the MIT license as source code and in binary form from https://github.com/virus-evolution/gofasta, as well as in binary form from Bioconda (Grüning *et al.*, 2018). It is designed for use on Unix-like (Linux and Mac OSX) operating systems. Where appropriate, it defaults to reading input from standard in (stdin) and writing output to standard out (stdout), so that commands can be incorporated into shell pipelines; in order to deal with large datasets, data are preferentially streamed, rather than loaded into memory. gofasta incorporates bíogo (Kortschak *et al.*, 2017) and makes use of the bitwise coding scheme for nucleotides by Emmanuel Paradis (Paradis *et al.*, 2004). Its main functionality is outlined below, including some brief use cases. Further documentation is available at https://github.com/virus-evolution/gofasta.

## 2. Functionality

### 2.1 Sam to fasta format conversion

Phylogenetic analysis, as is commonly performed in genomic epidemiological contexts (reviewed in Gardy and Loman, 2018), requires that sequences be aligned to each other. The accepted gold standard is multiple sequence alignment, but these methods are computationally expensive (reviewed in Edgar and Batzoglou, 2006), so are intractable for very large numbers of sequences and/or when fast turnaround times are required. When sequences are closely related, as is the case currently with SARS-CoV-2 for example, multiple pairwise alignments to a common reference sequence offer an efficient approximation. This technique is widely used in current analysis pipelines and software (Aksamentov *et al.*, 2021; Moshiri, 2021; Moustafa and Planet, 2021; O’Toole *et al.*, 2021a). gofasta provides functions to convert sequence alignment/map (sam) format files (Li *et al.*, 2009) representing pairwise alignments between assembled virus genomes including those output by minimap2 (Li, 2018) to fasta format (https://www.ncbi.nlm.nih.gov/genbank/fastaformat/). This is the pipeline that has been used by Pangolin (O’Toole *et al.*, 2021a) and the UK’s daily SARS-CoV-2 processing pipelines (https://github.com/COG-UK) during the SARS-CoV-2 pandemic to date. As an example, the current Pangolin pipeline for aligning SARS-CoV-2 sequences is equivalent to: 

minimap2 –a –x asm20 --score–N=0 --sam–hit–only --secondary=no reference.fasta unaligned.consensus. fasta –o aligned.sam

gofasta sam toMultiAlign –s aligned.sam --start 266 --end 29674 --pad –o aligned.fasta

In this routine, the alignment is trimmed to set reference coordinates using the --start and --end flags and the trimmed regions are replaced with Ns to preserve reference length with the --pad flag. These options may be useful, for example, to mask out untranslated regions in the case that they are more prone to errors due to lower sequencing coverage when using tiled amplicon schemes (Quick *et al.*, 2017), or to restrict downstream analyses to specific portions of the genome.

**Fig. 1.**
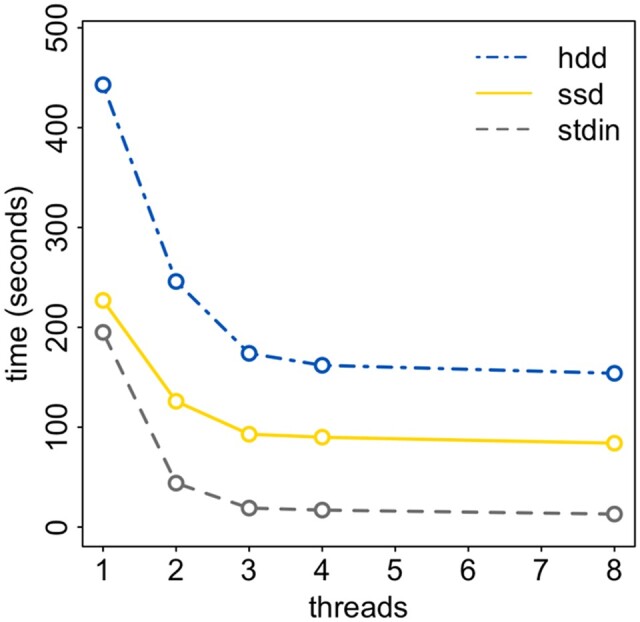
The additional time taken to convert a sam-format alignment of 1 million SARS-CoV-2 genomes to fasta format. Times are given for reading and writing from/to a rotating hard disk drive (dot-dashed line), from/to a solid state drive (solid line) and the additional time above minimap2’s runtime needed to write the fasta file to a hard disk drive while reading the sam file from standard in (dashed line). In each case, the sam file was generated on a server with 40 logical CPUs by running minimap2 with 32 threads and was ∼28 GB in size. gofasta was run with different numbers of threads (represented on the x-axis)

Equivalently, one can issue: 

minimap2 –t37 –a –x asm20 --score–N=0 --sam–hit–only --secondary=no reference.fasta unaligned.consensus.fasta | gofasta sam toma –t3 --start 266 --end 29674 --pad > aligned.fasta

which has the advantage that the fasta file is written concurrently and no intermediate sam file is written to disk, saving time and space. Timings for file format conversion of 1 million SARS-CoV-2 genomes are presented in Figure 1.

In the routine above insertions relative to the reference are omitted from the converted pairwise alignments so that all sequences in the fasta-format representation are the same (reference) length. gofasta also provides a routine (toPairAlign) that will convert a sam file to pairwise alignments in fasta format (one file per sequence) which include insertions relative to the reference and the reference sequence itself by default.

### 2.2. Genetic distances and searching for nearest neighbours

gofasta provides two routines which are designed to aid outbreak investigations by searching for neighbouring sequences by genetic distance. Both are optimized for comparing a smaller set of query sequences against a larger set of target sequences: the focal query sequences are loaded into memory and the background dataset of target sequences is streamed from disk, so the target file can be arbitrarily large.

The first routine, closest, takes alignments in fasta format as input and implements three standard distance measures for pairwise comparisons, two of which are uncorrected for multiple hits (the number of nucleotide differences, and the number of nucleotide differences per site) and one of which is a corrected evolutionary distance (TN93; Tamura and Nei, 1993). The user has the option to return the closest *n* neighbours or all neighbours under a set distance *d* for each query sequence. Catchments are either returned as lists of target neighbours, sorted by distance, with ties broken by genome completeness, or can be printed in long form with the distance between each query–target neighbour pair written.

The second routine, updown, implements an uncorrected distance measure (the number of nucleotide differences). Sequences must be pre-aligned, then are reduced to the set of ATGC single nucleotide polymorphisms (SNPs) by which they differ by from a common reference sequence, which is interpreted as being ancestral. Patterns of derived SNPs are used to infer the phylogenetic relationships between sequences (Fig. 2 and Fig. S1 in O’Toole et al., 2021b). For low-diversity datasets, this routine has the potential to be faster than traditional distance measures, because it avoids the computational cost of comparing every alignment position. Finding the 1000 closest genetic neighbours for a single query among a target of 10.5 million SARS-CoV-2 sequences takes 2 min and 40 s on a desktop computer with Intel processors and a solid-state hard drive. The equivalent command using closest and TN93 distance takes 8 min and 10 s. Peak memory usage for both routines was less than 10 MB for these examples.

### 2.3 Mutation calling

gofasta provides a routine (snps) for describing all individual nucleotide differences between a set of aligned sequences and a single reference sequence. It also provides routines to describe insertion, deletion, amino acid and nucleotide mutations from pairwise alignments in sam format (sam variants) or a multiple sequence alignment in fasta format (variants), given a reference sequence (or its identity in the multiple sequence alignment) and an annotation of the protein-coding content of the reference genome. Mutations are either annotated per-genome or can be aggregated, to provide frequencies per input file. In the latter case, an optional threshold can be applied to return only mutations that are present above a given frequency.

As a brief example, it would be possible to find the frequencies of all the amino acid changes at residue 681 in the Spike gene, and the nucleotide changes underlying them, from the sample of SARS-CoV-2 sequences in aligned.fasta like: 

gofasta variants --msa aligned.fasta --annotation MN908947.gb --aggregate --append-snps | grep "^aa:S:P681"

aa:S:P681H(nuc:C23604A),0.004000000

aa:S:P681R(nuc:C23604G),0.983000000
